# Feasibility of quantitative perfusion CMR in patients with poor left ventricular function

**DOI:** 10.1186/1532-429X-15-S1-E60

**Published:** 2013-01-30

**Authors:** Eva Sammut, Niloufar Zarinabad, Zhong Chen, Reza Razavi, Eike Nagel, Amedeo Chiribiri

**Affiliations:** 1 Kings College, London, UK

## Background

First pass perfusion CMR is a well-validated technique for the detection of inducible myocardial ischaemia. However, limited data are available on the feasibility of first pass perfusion CMR and quantitative deconvolution analysis in the context of reduced LV function.

The aim of this study was to assess effects of reduced LV function and LV dilatation on the characteristics of signal in the left ventricle (arterial input function, AIF) and in the myocardium (MSI), and to test the feasibility of quantitative perfusion analysis with Fermi deconvolution in this cohort of patients.

## Methods

We retrospectively identified fifteen consecutive patients who had undergone perfusion CMR in the presence of impaired LV function (left ventricular ejection fraction (LVEF) <50%) at our centre. Five healthy volunteers were enrolled in the study as control subjects. Perfusion data were obtained using a pre-bolus technique and 0.075 mmol/kg Gadobutrol (Gadovist, Schering, Germany) injected at 4ml/minute followed by a 20 ml saline flush. Images were acquired on a Philips Achieva 3T (TX) system, equipped with a 32-channel cardiac phased array receiver coil (Philips, Best, Netherlands). First-pass perfusion images were acquired using a saturation recovery gradient echo method (TR/TE 3.0 ms/ 1.0 ms, flip-angle 15°; effective k-t SENSE acceleration 3.8, spatial resolution 1.2x1.2x10 mm, saturation-recovery delay 120 ms).

The following parameters were evaluated from stress perfusion series in order to assess the quality of the data and assess the effect of abnormal cardiac structure and function: time to peak, peak value and maximum slope of the AIF and of the MSI. Quantitative analysis was performed by Fermi deconvolution.

## Results

Detailed results are reported in Table. The left ventricular (LV) systolic function was lower, with higher end-diastolic (LV EDV) and end-systolic volumes (LV ESV) in the patient compared with the normal group.

**Table 1:** Showing values across patient and control group

	Patient group	Control group	p value
LVEF (%)	40.1 (SD 9.4)	67.0 (SD 3.7)	<0.0001
LV EDV (ml)	108.3 (SD 34.0)	75.6 (SD 7.8)	0.0030
LV ESV (ml)	67.1 (SD 31.1)	25.2 (SD 4.1)	0.0001
Peak AIF	389.4 (SD 328.2)	1239.7 (SD 637.8)	0.0379
Peak MSI	113.5 (SD 83.4)	358.4 (SD 199.4)	0.0497
Peak AIF / peak MSI	3.6 (SD 1.4)	3.7 (SD 0.7)	0.9054
Time to peak LVAIF (seconds)	9.1 (SD 3.2)	6.1 (SD 0.8)	0.0052
Time to peak MSI (seconds)	12.1 (SD 4.5)	7.6 (SD 1.0)	0.0025
Time to peak MSI / time to peak AIF (seconds)	3.1 (SD 3.7)	1.5 (SD 0.5)	0.1250

Both the AIF and the MSI were reduced in peak amplitude, maximum upslope and time to peak in patients compared with controls. However, the ratio between the peak of AIF and MSI and the time to peak of the AIF and MSI was constant between patients and controls, suggesting the feasibility of deconvolution quantitative analysis on both groups.

Fermi deconvolution showed a trend towards an overall lower perfusion rate in normal segments in the patients group (2.2±1.2 ml/g/min) compared with controls (2.7±0.8; p=0.2) however non-significant. Reduced perfusion rate was found in visually abnormal segments in patients (1.6±0.8; p=0.03).

## Conclusions

Our results show that reduced cardiac function modulates the characteristics of both the AIF and MSI. However, the relationship between AIF and MSI remains unaffected. Fermi deconvolution analysis appears applicable to both groups, supporting the feasibility of first pass perfusion CMR as a clinical tool in patients with reduced LV function.

## Funding

None.

**Figure 1:**
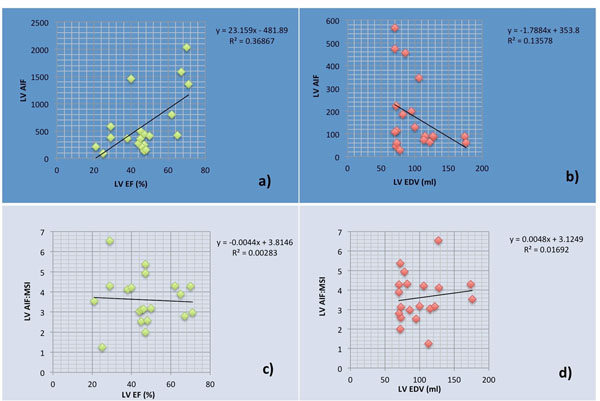
a) LVEF vs peak LV AIF, b) LV EDV vs LV AIF, c) LV EF vs peak LVAIF/peak MSI, d) LV EDV vs peak LVAIF/peak MSI

